# P301: The blood exposure accidents to hospital regional of Tenkodogo

**DOI:** 10.1186/2047-2994-2-S1-P301

**Published:** 2013-06-20

**Authors:** J Zoungrana, A SO

**Affiliations:** 1 RIPAQS, Burkinabe Association for Improving Patient Safety, Burkina Faso; 2 CHR Tenkodogo Tenkodogo, Ministry of Health, Ouagadougou, Burkina Faso

## Introduction

Accidental blood exposure (AES), whether occurring during professional activities or not, is defined by contact with blood or blood-containing liquid at a prick with a needle, a cut with a sharp objects or by contact with blood or contaminated liquid from a wound, non-intact skin or mucous membranes.

To date, very few studies have been conducted in the field of AES in Burkina Faso.

## Objectives

- To study the mechanisms of occurrence of AES at CHR Tenkodogo,

- To identify the main causes of AES,

- To identify occupational groups at risk of AES,

- To evaluate the support provided by the hospital for victims of AES.

## Methods

- 1 week, cross-sectional descriptive study (March to 10 March 2011).

- Data collection performed using a self-administered questionnaire.

## Results

60% of patients reported no exposure to blood. In 90.9% of cases of blood exposure accidents are occurring in health care administration. Needle-stick injury is the most prevalent in a proportion of about 50%. Most needle-stick injuries involved the fingers. In 86.4% of cases, the contaminating liquid was blood. The general surgery service is by far the one who pays the brunt of exposure to blood in a proportion of 34.7% (19.6% in surgery and 15.2% at PO block) followed générale15 Medicine Department, 22% of 82.3% of victims AES. AES did not meet protocol support 18.8% of those exposed have received ARV treatment 25.6% of cases of BSE reported n have received no support.

## Conclusion

It is important for healthcare facilities to implement monitoring of AES to evaluate prevention policies.

## Disclosure of interest

None declared.

